# Functionalized Imidazolium Ether-Free Polymer Backbones with Ion Transport Channels and Catalytic Activity

**DOI:** 10.1021/acsmaterialsau.4c00154

**Published:** 2025-03-27

**Authors:** Bryan A. Corzo, Hugo Hernández-Martínez, Eugenia Josefina Aldeco-Pérez, Jorge Cárdenas, Víctor Lara, Lilian I. Olvera

**Affiliations:** † Instituto de Investigaciones en Materiales, Universidad Nacional Autónoma de México, Apartado Postal 70-360, CU, Coyoacán, 04510 Ciudad de México, México; ‡ Centro de Investigación y Desarrollo Tecnológico en Electroquímica S.C., Parque Tecnológico Querétaro, Sanfandila, Pedro Escobedo C.P. 76703, Querétaro, México; § Instituto de Química, Universidad Nacional Autónoma de México, Apartado Postal 70-360, CU, Coyoacán, Ciudad de México 04510, México; ∥ UAM-I, Av. Michoacán y Purísima, Iztapalapa 09340 México City, México

**Keywords:** superacid, anion exchange membranes, electrochemical devices, organometallic polymer, *N*-heterocyclic carbene

## Abstract

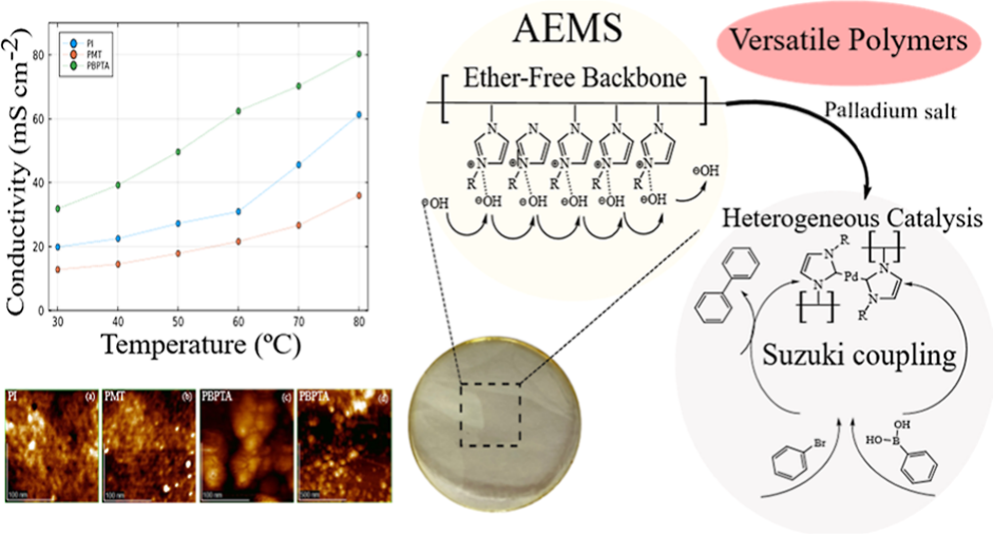

Novel ether-free bond polymer backbones were synthesized through polycondensation in a superacid medium by using *p*-terphenyl and 4-(1*H*-imidazol-1-yl)benzaldehyde. The presence of imidazolium groups enabled further modifications through a highly efficient nucleophilic substitution reaction introducing cationic sites essential for anionic transport. Characterization by NMR and FTIR analyses confirmed the structures and the complete functionalization of the base polymer. Critical properties for potential anion exchange membrane applications, including water uptake, ion exchange capacity, ion conductivity, morphology, and thermal and mechanical stabilities were investigated. Results indicated that these polymers form stable ion transport channels, with the formation of distinctive hydrophilic/hydrophobic microphase separation in the membranes observed through AFM, HR-TEM, and SAXS analyses. This structural configuration of the membranes exhibited high hydroxide conductivities of 61.33 and 80.33 mS/cm at 80 °C for 1AIM (quaternization with iodomethane) and 1ABPTA (quaternization with (3-bromopropyl)trimethylammonium bromide), respectively, with a thermal stability up to 240 °C, underscoring their suitability for electrochemical applications. Additionally, an organometallic polymer was successfully synthesized from the 1ABPTA polymer due to the presence of an imidazolium salt, *N*-heterocyclic carbene (NHC) ligand precursor. SEM images displayed the homogeneous distribution of metal atoms, and XPS spectra confirmed the formation of the C–M bond. The material obtained was utilized as a heterogeneous catalyst in a C–C Suzuki–Miyaura coupling reaction, achieving catalytic conversion percentages of 70% and 60% for the first and second cycles, respectively.

## 1 Introduction

Nowadays, anion exchange membranes (AEMs) are used in various electrochemical devices, such as fuel cells, electrolyzers, and redox flow batteries.^1−4^ Particularly, AEMs are polymers characterized by the presence of positively charged functional groups^4,5^ and have attracted great interest due to the promise of low-cost sources of renewable energy for the future.^3,6^ However, the membranes that have undergone the most development and have been most widely commercialized to date are proton exchange membranes (PEM), specifically Nafion, due to their good chemical and mechanical stability. Nevertheless, the complexity of the synthesis and high costs are issues that have led scientific convenience to opt for other polymeric membranes, like sulfonated nonfluorinated hydrocarbon polymers, as poly(arylene ether ketone)s (PAEKs) or poly(arylene ether sulfone)s (PAESs) that combine lower cost and display high chemical and mechanical stabilities.^5,7^ Additionally, the acidic conditions in which PEMs operate generate slow kinetics for the reduction of oxygen and therefore require the use of platinum-based catalysts, increasing the costs of electrochemical devices.^4,8−10^ In this sense, AEMs, which, unlike PEMs, allow for the reduction of oxygen at the cathode under alkaline conditions in a faster and more efficient manner.^9,11^

Nevertheless, there are different problems and challenges associated with the development of AEMs since materials with high chemical stability at high pH levels as well as high conductivities are required. One of the strategies used to achieve high ionic conductivity is by increasing the ion exchange capacity (IEC) of the membrane (IEC); however, this results in excess of water uptake (WU), leading to a drastic decrease in mechanical properties.^12−14^ Currently, there are different types of AEMs, and the most common are those based on polysulfones^15−19^ and poly(ethylene oxide)s.^20^ However, these types of materials have a limitation since they have ether bonds in the main chain that are susceptible to degradation.^3,4^ Therefore, the development of new polymer-forming reactions that allow obtaining materials with high chemical, mechanical, and thermal stabilities is essential. On the other hand, polymers without ether bonds in the main chain have been reported, obtained through polycondensation reactions for its use as AEMs, allowing the development of new structures, achieving an adequate balance between hydration, conductivity, and thermal, mechanical, and chemical stability.^3,21−24^ Most of these membranes have quaternary ammonium groups or piperidinium groups in their structure.^22−24^ With the aim of increasing the stability of the cationic group and having alternatives, guanidinium, phosphonium, and imidazolium groups have been studied; however, stable guanidinium or phosphonium groups are costly. Imidazolium groups have gained importance due to their potential use as stable cationic groups in AEMs at a lower cost.^2,11,25−29^ In a recent report, Zhang and collaborators^30^ developed polyelectrolytes introducing the highly alkaline stable imidazolium cation PhIM [PF6], modified by the propylbenzene group on the position of N1/N3, of the imidazole group, achieving highly alkaline stable poly(arylene-imidazolium) polymers with the imidazolium group in the main chain. Incorporating these heterocyclic groups into the polymer structure greatly enhances the versatility of these materials for different applications. This is primarily due to the presence of heteroatoms such as nitrogen, which, with its available lone pairs of electrons, can form bonds with various side chains, thereby imparting a wide range of properties. In particular, imidazolium salts are precursors of the so-called *N*-heterocyclic carbenes, which are of great interest in organometallic chemistry due to their strong ligand sigma donation characteristics and their ability to stabilize a wide variety of transition metals.^31,32^ Here, the synthesis of polymers free of ether linkages in the main chain through the polyhydroxyalkylation reaction catalyzed by superacid of the commercial monomer 4-(1*H*-imidazol-1-yl)benzaldehyde and *p*-terphenyl is demonstrated, resulting in novel polyelectrolytes with imidazole groups as the lateral chain, favoring phase segregation and leading to an increase in ionic conductivity. The reaction of one of these polyelectrolytes with palladium acetate was conducted to obtain an organometallic polymer and its catalytic activity was evaluated in a Suzuki–Miyaura C–C coupling reaction.

## 2 Experimental Section

### 2.1 Materials

All of the starting materials were obtained from Aldrich. The solvents and catalysts compounds as dichloromethane (CH_2_Cl_2_), *N*-methyl-2-pyrrolidone (NMP), ethyl acetate (EtOAc), dimethyl sulfoxide (DMSO), dimethylacetamide (DMAc), dimethylformamide (DMF), chloroform (CHCl_3_), trifluoromethanesulfonic acid (TFSA), trifluoroacetic acid (TFA), and methyl trifluoromethanesulfonate (MeOTf) were distilled prior to used, while *p*-terphenyl, (3-bromopropyl)trimethylammonium bromide, tetrahydrofuran (THF), iodomethane, 4-(1*H*-imidazol-1-yl)benzaldehyde, and potassium carbonate were used as received.

### 2.2 Polymer Synthesis

#### 2.2.1 Base Polymer **(1A)**

**(1A)** was synthesized via a polyhydroxyalkylation reaction in superacid media in a nonstoichiometric condition. In a flask containing a mechanical stirrer, *p*-terphenyl (2.7653 g, 12.01 mmol) and 4-(1*H*-imidazol-1-yl)benzaldehyde (2.6935 g, 15.64 mmol) were added to dichloromethane (11 mL). The solution reaction was placed into an ice bath and trifluoroacetic acid (1.5 mL) followed by trifluoromethanesulfonic acid (7.5 mL) was added slowly. The reaction was carried out at room temperature under stirring for 3 h resulting in a viscous homogeneous dark-blue solution which was subsequently poured and precipitated into methanol. The obtained white fibrous polymer material was washed in cold methanol and dried overnight in air. The protonated polymer was fully soluble in NMP, DMF, DMAc, and DMSO. To deprotonate the polymer, a reprecipitation was conducted using *N*-methyl-2-pyrrolidone (NMP) and precipitated in a solution of NaOH (1 M), washed several times with water until neutral pH was achieved, and dried in air overnight, resulting in 4.5498 g (10.96 mmol) of white fiber polymer (91.4% yield).

#### 2.2.2 Imidazolium-Functionalized Polymers

Postsynthesis chemical modification reactions of the base polymer (1A) were carried out to incorporate quaternary ammonium groups into the polymer backbone. 1A was chemically modified with iodomethane (IM), methyl trifluoromethanesulfonate (MT), or (3-bromopropyl)trimethylammonium bromide (BPTA) to produce three different polyelectrolytes (PE).

#### 2.2.3 Quaternization with Iodomethane (1AIM)

The modification reaction of 1A with iodomethane was carried out by dissolving 1 g of polymer 1A in 10 mL of NMP and adding 1.2373 g (8.717 mmol) of iodomethane. The mixture was stirred for 5 days at room temperature. Afterward, the solution was poured into ethyl acetate to obtain brown fibers. The obtained material was washed in warm ethyl acetate for at least 6 h and then reprecipitated from NMP into ethyl acetate and washed several times before drying. The resulting pure fibers (1.3856 g, 97% yield) displayed an inherent viscosity of 1.83 dL g^–1^.

#### 2.2.4 Quaternization with Methyl Trifluoromethanesulfonate (1AMT)

One gram aliquot of polymer 1A was dissolved in 10 mL of NMP and 1.4382 g (8.764 mmol) of methyl trifluoromethanesulfonate was then added. The mixture was stirred for 5 days at room temperature. Afterward, the solution was poured into ethyl acetate to obtain white fibers. The obtained material was washed in warm ethyl acetate for at least 6 h and then reprecipitated from NMP into ethyl acetate and washed several times before drying. The resulting pure fibers (1.4225 g, 97% yield) displayed an inherent viscosity of 1.59 dL g^–1^.

#### 2.2.5 Quaternization with (3-Bromopropyl)trimethylammonium Bromide (1ABPTA)

The modification reaction of 1A with (3-bromopropyl)trimethylammonium bromide was carried out by dissolving 1 g of polymer 1A in 10 mL of NMP and adding 2.1992 g (8.426 mmol) of BPTA. The mixture was stirred for 5 days at room temperature. Afterward, the solution was poured into ethyl acetate to obtain a brown fiber. The obtained material was washed in warm ethyl acetate for at least 6 h and then reprecipitated from NMP into ethyl acetate and washed several times before drying. The resulting pure fibers (1.6691 g, 96% yield) had an inherent viscosity of 2.10 dL g^–1^. The ^1^H NMR spectra of PEs were used to corroborate 100% of the degree of functionality (complete modification).

### 2.3 Membrane Preparation

0.30 g of polyelectrolytes in Br^–^, I^–^, or ^–^OTf form was dissolved in 3 mL of DMSO, achieving 10% w/v of polymer. The solution was casted onto a glass Petri dish and dried at 70 °C for 72 h, obtaining membrane thicknesses of around 25 μm.

### 2.4 Characterization

^1^H and ^13^C NMR spectra were used to determine the molecular structures of the base polymer (1A) and polyelectrolytes (PEs) on a Bruker Avance 400 spectrometer using chloroform-*d* (CDCl_3_) and dimethyl sulfoxide-*d*_6_ (DMSO-*d*_6_) as solvents for 1A and PEs, respectively. Infrared spectra (IR) were recorded on a Thermo Scientific Nicolet iS10 FTIR. The inherent viscosities were determined using an Ubbelohde viscometer at 25 °C with solutions of 0.2% w/v of 1A and PE materials in CHCl_3_ and DMSO, respectively, using the following eq 1
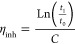
1where *t*_1_ and *t*_0_ are the fall times of the sample and blank, respectively, and *C* is the solution concentration. Thermal decomposition of the base polymer and polyelectrolytes was studied by thermogravimetric analysis (TGA) using a SDT Q600 Simultaneous Thermal Analyzer under a nitrogen atmosphere at a heating rate of 10 °C/min in a range between 25 and 800 °C.

The morphology of the polyelectrolyte membranes was observed using an atomic force microscope (AFM) on a JEOL JSPM-4210, also high-resolution transmission electron microscopy was carried out on a JEOL ARM-200F Cs-aberration-corrected microscope, and small-angle X-ray scattering (SAXS) was performed using a Kratky camera coupled to a copper anode tube whose K̅α radiation was selected with a nickel filter. The mechanical properties were recorded on a universal/tensile testing machine AGS-X with a tensile rate of 10 mm/min at 22 °C and 53% relative humidity.

IEC of the PEs membranes was determined using a classical Mohr method titration; membranes in halogenated form were dried at 60 °C for 24 h and weighed, and then the membranes were immersed in a 0.2 M solution of sodium nitrate (NaNO_3_) for 72 h. The remaining NaNO_3_ solution was titrated with a 0.01 M solution of silver nitrate (AgNO_3_) using potassium chromate (K_2_CrO_4_) as an indicator. The IEC values were calculated using the following eq 2
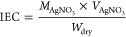
2where  is the concentration of AgNO_3_ solution,  is the volume in mL used in the titration, and *W*_dry_ is the weight of the dry membrane.

For the WU and swelling ratio (SR), the membranes were immersed in deionized water at 25 or 80 °C for 24 h, then the samples were removed from the water, and the surface water was removed with a wipe and immediately weighted and measured. WU and SR were calculated using the following eqs 3 and 4
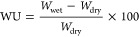
3
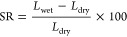
4where *W*_wet_, *L*_wet_ and *W*_dry_, *L*_dry_ are the weights and lengths of the wet and dry membranes, respectively. The hydration number (λ) represents the number of water molecules around each cationic position and can be calculated using eq 5
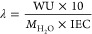
5where  is the molar mass of water (18.02 g/mol) and IEC is the ion exchange capacity in (mmol/g). The –OH ion conductivity of polyelectrolyte membranes in the fully hydrated sample was measured using a four-probe electrode cell and determined using eq 6
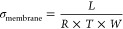
6where *L* is the distance between two inner Pt sensing wires (cm), *R* is the measured resistance (Ohm), *T* is the thickness of the membrane (cm), and *W* is the membrane width (cm). The *R* value was determined using impedance spectroscopy in a frequency range of 1–10^5^ Hz with an amplitude of 20 mV on a Biologic SP-50e potentiostat at different temperatures.

X-ray photoelectron spectroscopy (XPS) analyses were performed on an ultrahigh vacuum system Scanning XPS microprobe PHI 5000 VersaProbe II, with an Al Kα X-ray source (hν = 1486.6 eV) monochromatic with a 100 μm beam diameter and an MCD analyzer. XPS spectra were recorded at 45 °C to the normal surface in the constant pass energy mode E0 = 117.40 and 11.75 eV for the survey surface and high-resolution narrow scan, respectively. The peak positions were referenced to the background silver 3d_5/2_ photopeak at 368.20 eV, having a full width at half-maximum of 0.56 eV, and C 1s hydrocarbon groups at 285.00 eV.

## 3 Results and Discussion

### 3.1 Monomer Selection

Commercially available 4-(1*H*-imidazol-1-yl)benzaldehyde was used for polymer reactions with *p*-terphenyl, these monomers were selected due to their commercial availability with relatively low cost, the incorporation of multiring aromatic hydrocarbons increases the chemical stability and glass transition temperatures, and the incorporation of a heterocyclic ring, like imidazole, allows the alkylation of these groups making them versatile materials. The presence of imidazole in the polymer structure allows chemical postmodifications reactions to provide a wide range of structures with multiple properties and functionalities.

### 3.2 Polymer Synthesis and Characterization

**Scheme 1:**
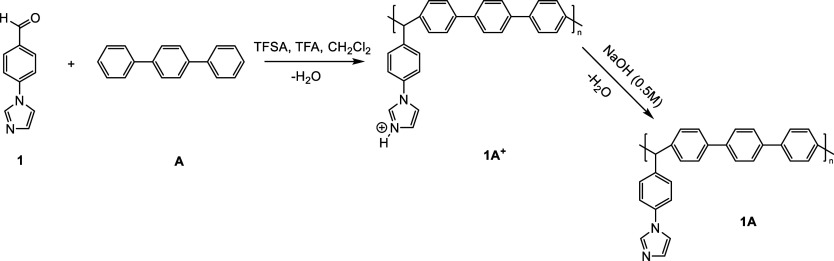
Polymerization of 4-(1*H*-Imidazol-1-yl)benzaldehyde and *p*-Terphenyl and Subsequent Deprotonation to Obtain 1A

The poly((*p*-tolyl)imidazoleterphenylene) (1A) was synthesized by a superacid-catalyzed polyhydroxyalkylation reaction (methodology developed at the “Universidad Nacional Autónoma de México”, (UNAM-route)) in a nonstoichiometric condition,^33−36^Scheme 1.

Generally, to obtain high molecular weights through polycondensation, strict control of stoichiometry between monomers is necessary; however, polyhydroxyalkylations have been reported using the nonstoichiometric effect,^37−39^ which consists of the addition of a slight excess of the carbonyl compound, achieving polymers with high or ultrahigh molecular weight in shorter reaction times, allowing the use of monomers without high degrees of purity. The reactions proceeded in Bronsted superacid, like trifluoromethanesulfonic acid, in mixture with dichloromethane and trifluoroacetic acid at room temperature, obtaining a high-molecular weight polymer^40,41^ (Table 1).

**Table 1:** Polymerization Result for the Polymer Obtained by Reaction of 4-(1*H*-Imidazol-1-yl)benzaldehyde and *p*-Terphenyl

ID	monomer concentration (mol L^–1^)	TFSA/ketone (mol mol^–1^)	reaction time (h)	yield (%)	inherent viscosity (dL g^–1^)
1A	0.6	5.4	3	92	2.2

**Scheme 2:**
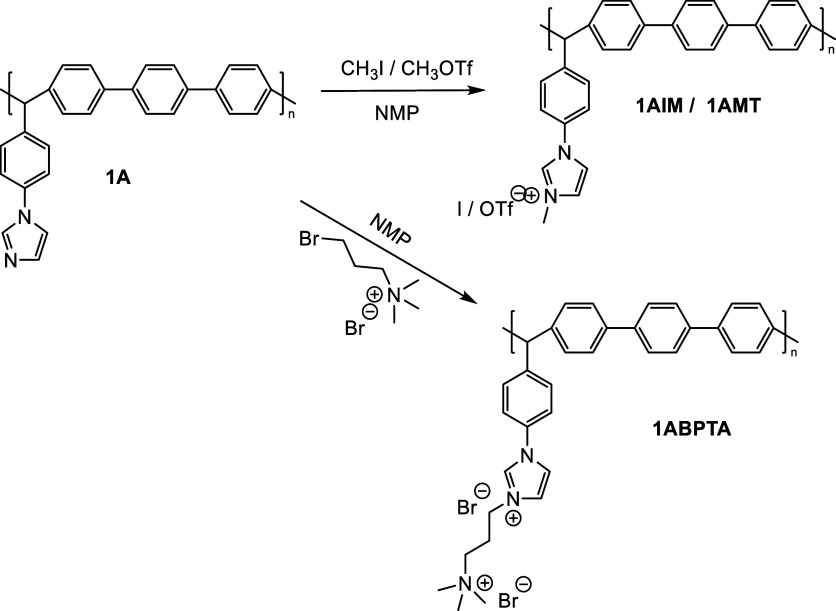
Synthesis for Solid Polyelectrolytes 1AIM, 1AMT, and 1ABPTA

To obtain polyelectrolytes (PE), an alkylation reaction of the 1A polymer with iodomethane (1AIM), methyl trifluoromethanesulfonate (1AMT), and (3-bromopropyl)trimethylammonium bromide (1ABPTA) was carried out (Scheme 2). Alkylation reactions were carried out with high functionalization degrees (100%) to generate a positive charge in the imidazole group. The results of the modifications are shown in Table 2.

**Table 2:** Modifications Reactions Results for Cationic Polymers

ID	base polymer (mmol)	modification reagent (mmol)	reaction time (days)	yield (%)	inherent viscosity (dL g^–1^)
1AIM	2.63	8.72	5	96	1.83
1AMT	2.61	8.76	5	85	1.59
1ABPTA	2.60	8.43	5	98	2.10

**Figure 1:**
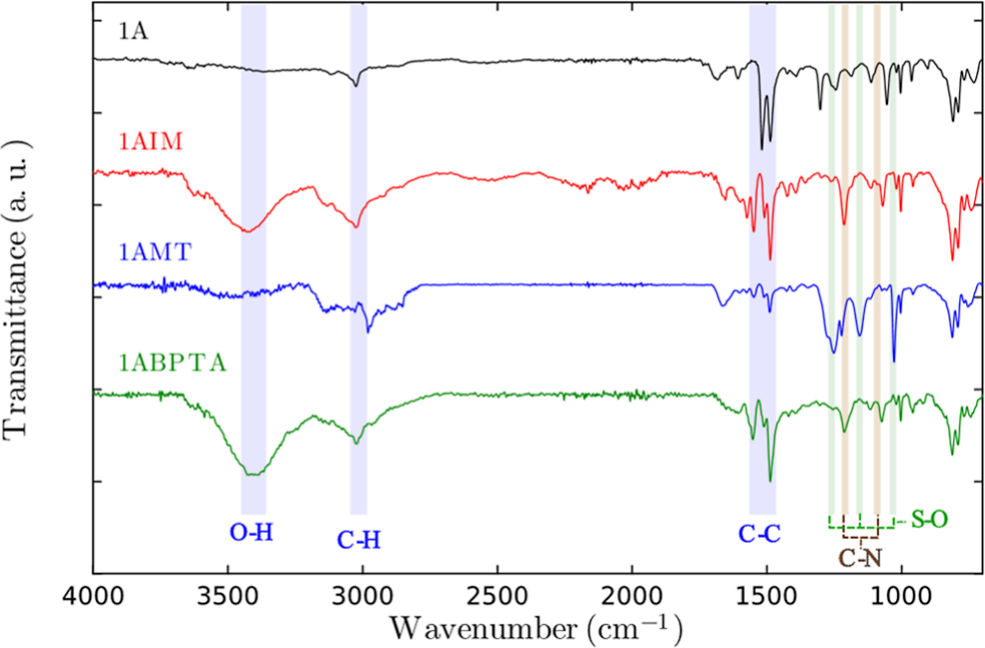
FTIR spectra for base polymer (1A) and polyelectrolytes 1AIM, 1AMT, and 1ABPTA.

All of the obtained polymers were characterized using FTIR and NMR techniques. FTIR analysis was used to confirm the total modification of the precursor polymer (1A). The FTIR spectra of 1A and polyelectrolytes (PEs) are shown in Figure 1. The bands around 3400 cm^–1^ are only present in the polyelectrolytes and correspond to the O–H stretching of water, due to the hydrophilicity that the quaternary groups confer to the polymer. The bands at 3010 and 2970 cm^–1^ can be assigned to C–H stretching of aromatic and aliphatic carbons, respectively. The bands between 1550 and 1475 cm^–1^ correspond to C=C stretching for the aromatic backbone. Signals at 1210 and 1091 cm^–1^ (brown lines) correspond to the stretching vibrations of the C–N bond in the imidazole group and the alkyl chain, respectively.^42^ Bands at 1260 and 1032 cm^–1^ (green lines) correspond to SO_3_ asymmetric and symmetric stretching modes and the band at 1156 cm^–1^ (green line) corresponds to the asymmetric CF_3_ bond of the trifluoromethanesulfonate group in 1AMT.^43^

**Figure 2:**
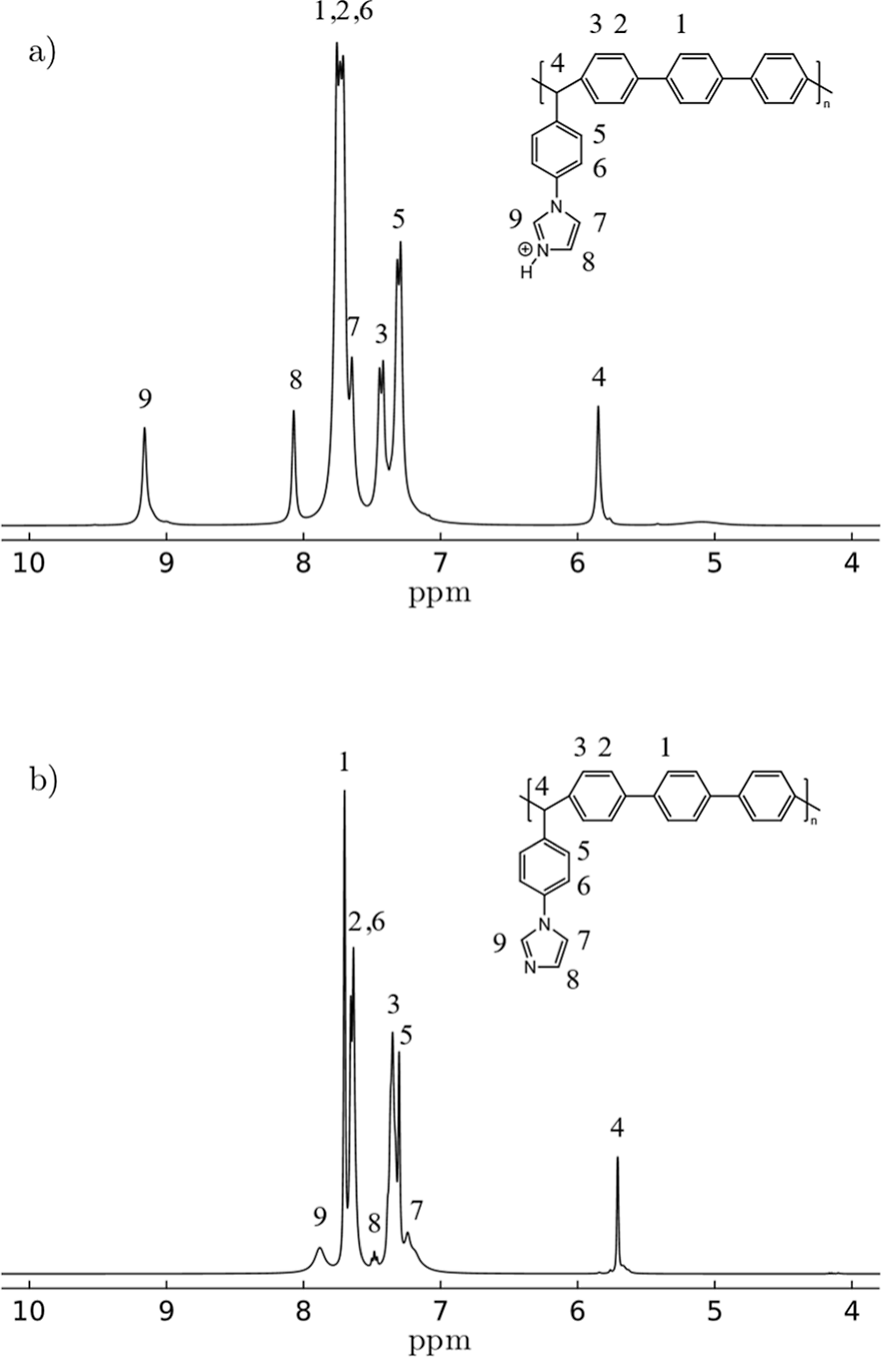
^1^H NMR spectra of protonated base polymer 1A^+^ (a) and base polymer 1A (b).

The high solubility of the precursor polymer, as well as the polyelectrolytes obtained, allowed NMR studies to be carried out to determine the structure of the synthesized materials. In the proton NMR spectrum, a high regioselectivity could be observed since only para substitutions could be identified in the aromatic fragment without the presence of irregularities. Since the polycondensation reaction is carried out in a superacid medium, a protonated material (**1A**^**+**^) was obtained by observing the proton signals of the imidazolium group at 9.1, 8.05, and 7.65 ppm (Figure 2a). After treatment with NaOH 1 M, the signals of the imidazole group shifted to high fields at 7.9, 7.5, and 7.25 ppm, respectively (Figure 2b). Figure S1 shows the 2D COSY ^1^H NMR spectrum of protonated base polymer 1A^+^, ensuring correct signal assignment.

**Figure 3:**
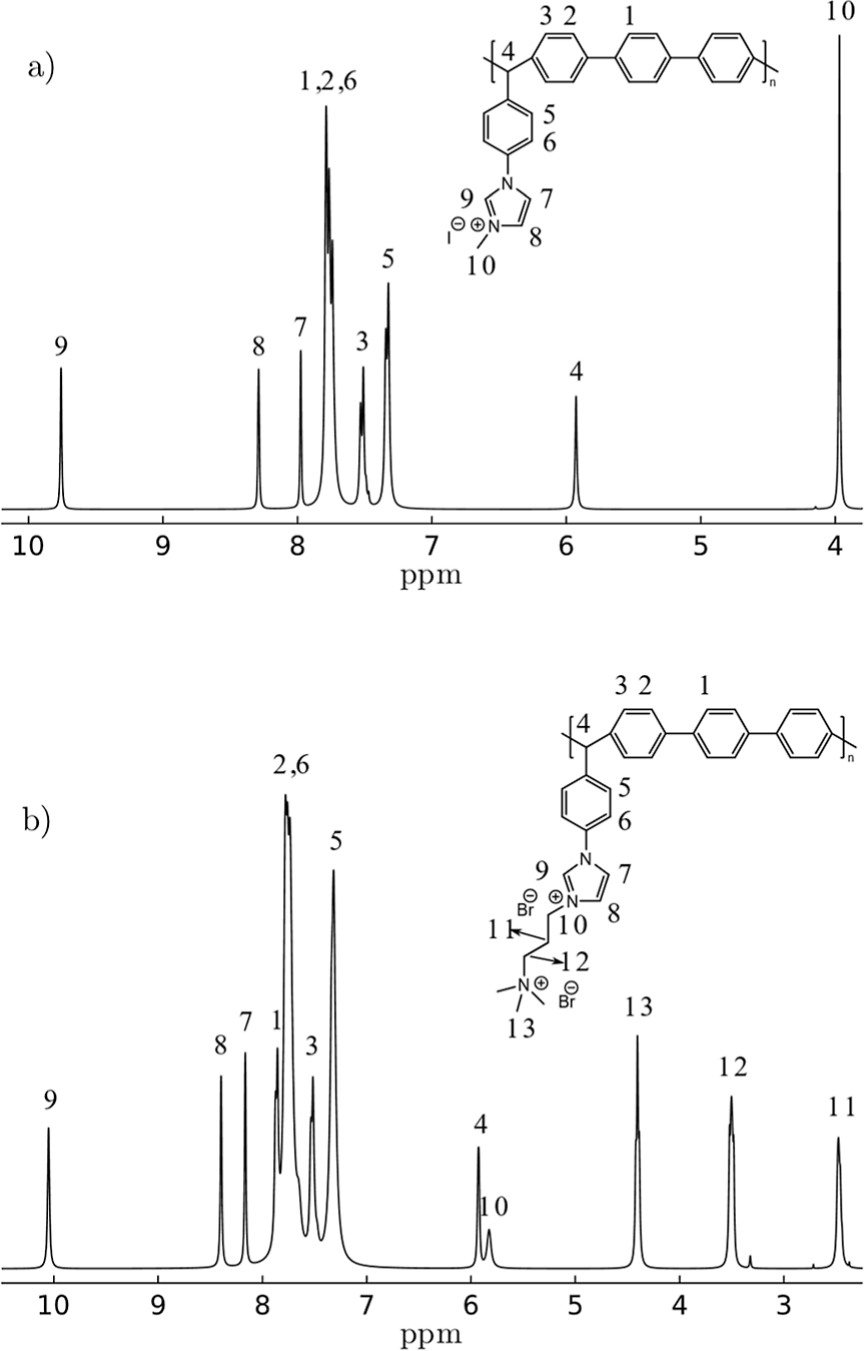
^1^H NMR spectra of polyelectrolytes (a) 1AIM and (b) 1ABPTA.

Polymer 1A was quaternized using iodomethane, methyl trifluoromethanesulfonate, and (3-bromopropyl)trimethylammonium. Figure 3 shows the NMR proton spectra of polyelectrolytes that confirm incorporation of the modification reagent. In the case of polyelectrolytes (1AIM and 1AMT), Figures 3a and S2, the proton signals belonging to the imidazolium group at 9.8, 8.1, and 7.95 ppm are present, and a new signal appears at 4 ppm corresponding to the methyl group. For the modification with (3-bromopropyl)trimethylammonium bromide (Figure 3b), the same pattern for imidazolium group signals was observed at 10, 8.25, and 8.1 ppm, and four new signals appeared at 5.8, 4.25, 3.4, and 2.5 ppm corresponding to protons of the aliphatic chain. In all cases, the signals between 7.0 and 8.0 ppm correspond to the aromatic protons in the main chain, and the signal at 5.9 ppm corresponds to aliphatic protons from the benzaldehyde group.

### 3.3 Polymer Properties

Table 3 shows the qualitative solubility properties of the materials obtained. It can be observed that precursor polymer 1A possesses a high solubility in chlorinated solvents due to its high backbone hydrophobicity. After modifications to incorporate cationic groups, PEs exhibit a complete solubility in polar solvents such as DMSO and DMF, due to an increase in the hydrophilicity conferred by cationic groups.

**Table 3:** Polymers Solubility^a^ and Thermal Properties

			solvent						
polymer	Td (°C)	char yield, 800 °C (%)	CHCl_3_	DMF	DMAc	DMSO	THF	NMP	acetone
1A	550	69.5	+	–	+	–	–	+	–
1AIM	478	50.1	–	+	+	+	–	+	–
1AMT	452	46.7	–	+	+	+	–	+	–
1ABPTA	448	46.1	–	+	–	+	–	+	–

a + Soluble, – insoluble.

**Figure 4:**
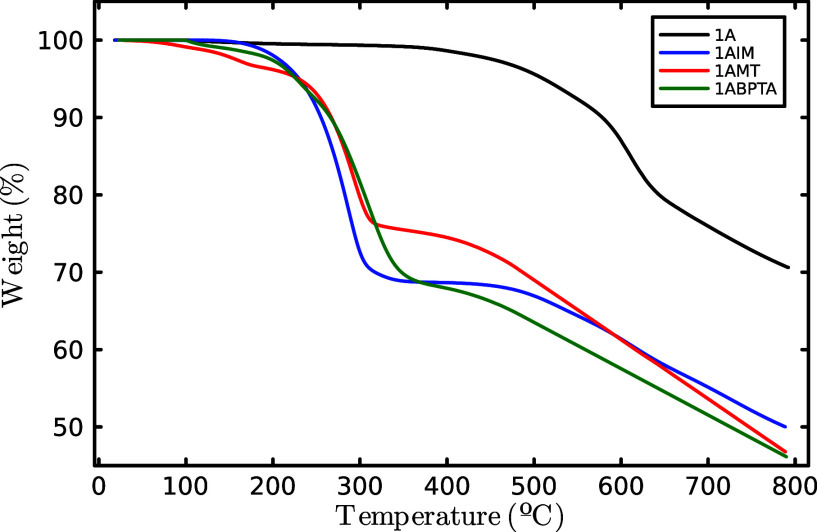
TGA thermograms of base polymer (1A) and polyelectrolytes.

Resistant, flexible, and transparent AEMS were prepared by dissolving the polyelectrolytes 1AIM, 1AMT, and 1ABPTA obtained in DMSO 10% w/v and casted onto a Petri dish. The most desired properties for applications as AEMs in electrochemical devices such as fuel cells, electrolyzers, and flux redox batteries are good thermal, chemical, and mechanical stability, low SR and WU, high alkaline stability, low ionic resistance, and sufficient long-term resistance at elevated temperatures and pH conditions.^44,45^ TGA in a N_2_ atmosphere was conducted to evaluate the thermal decomposition of base polymer and polyelectrolytes. Base polymer 1A showed only one step thermal decomposition at 550 °C. However, polyelectrolytes showed a two-step thermal decomposition pattern and initial moisture loss around 100 °C, due to the presence of cationic groups that absorb moisture from the environment. The loss in weight in the first step from 240 to 245 °C is presumably due to the decomposition of the side chain and the cationic groups, and the second step above 400 °C could be ascribed to the decomposition of the polymer backbone (Table 3 and Figure 4). The decomposition of the PEs started from 240 °C, as compared to operating temperatures for most of the electrochemical energy systems, around 100 °C for the AEMFC; these materials could be a viable option for practical applications.

**Figure 5:**
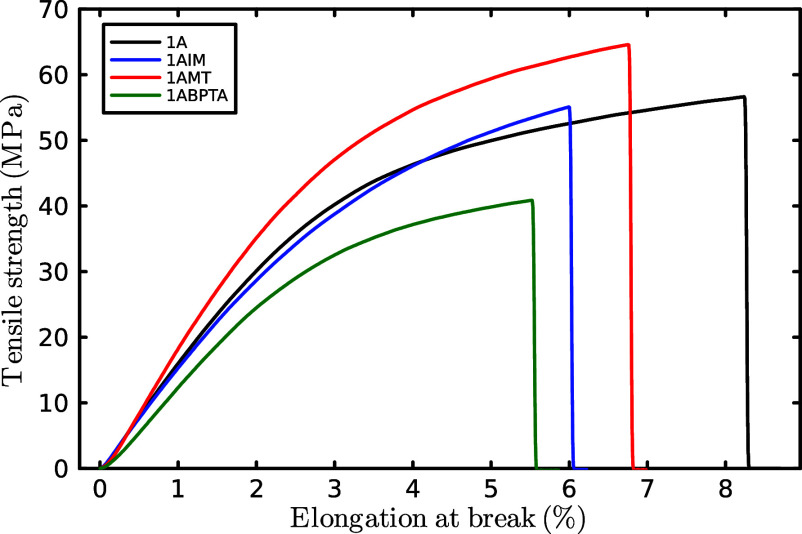
Mechanical properties of polycationic membranes.

Figure 5 shows the tensile strength values ranging between 40.9 and 64.6 MPa for the obtained PEs which is slightly higher than those previously reported for similar membranes.^46−48^ This is probably due to the presence of the *p*-terphenyl group in the polymer backbone and all of the aromatic rings present, conferring a higher mechanical and thermal stability. The elongation at break values are in the range between 5.5% and 8.2%, and Young’s modulus is 1.66, 1.54, 1.82, and 1.36 GPa for 1A, 1AIM, 1AMT, and 1ABPTA, respectively. The excellent mechanical strain data indicate that the polymers synthesized in this study are ductile, resistant, flexible enough, and suitable for their assembling within membrane electrodes MEA and further testing in electrochemical devices such as fuel cells or electrolyzers.^48−50^

WU, SR, IEC, and ion conductivity (σ) are displayed in Table 4. These properties are critical parameters to achieve good performance of AEMs in electrochemical devices. It has been determined that one of the key factors for achieving high anionic or hydroxide ion conductivity is a relatively high IEC value and a good membrane hydration (WU).^51,52^ IEC measures the density of active cationic groups in the membrane, which significantly affect the WU and ion conductivity; a higher value of IEC was accompanied by an increase in ion conductivity. IEC was measured by using a Mohr titration method. As expected, IEC values increased with an increase of cationic groups per monomer in the AEMs, obtaining values of 1.88 and 1.06 (one cationic group per monomer unit) for 1AIM and 1AMT, respectively, and 2.84 (two positive charges per monomer) for 1ABPTA. The difference between the IEC obtained in 1AIM and 1AMT polyelectrolytes could be associated with the counterion (iodide and triflate ions, respectively), and a significant decrease of IEC in 1AMT could be observed, probably due to much slower exchange kinetics in the case of triflates as compared to iodides.

**Table 4:** Properties of AEMs

		WU (%)		SR (%)			
polymer	IEC (mmol/g)	30 °C	80 °C	30 °C	80 °C	σ* (mS cm^–1^)	λ
1AIM	1.88	11.11	19.05	2.70	2.78	61.33	5.88
1AMT	1.06	12.05	16.67	2.63	2.63	35.97	8.74
1ABPTA	2.84	18.52	20.00	2.94	5.88	80.31	3.91

The WU depends on different factors, including the polymer backbone and the functional groups that make a membrane hydrophobic or hydrophilic. The WU increases with increasing IEC; however, a high IEC reflects excessive WU and high SR, drastically decreasing the mechanical properties. In this way, an increase in the ion conductivity is correlated with an increase in WU, until excessive values result in an increased SR, leading to a poor dimensional stability and dilution of the ionic groups in the membrane causing a reduction in ion conductivity.

**Figure 6:**
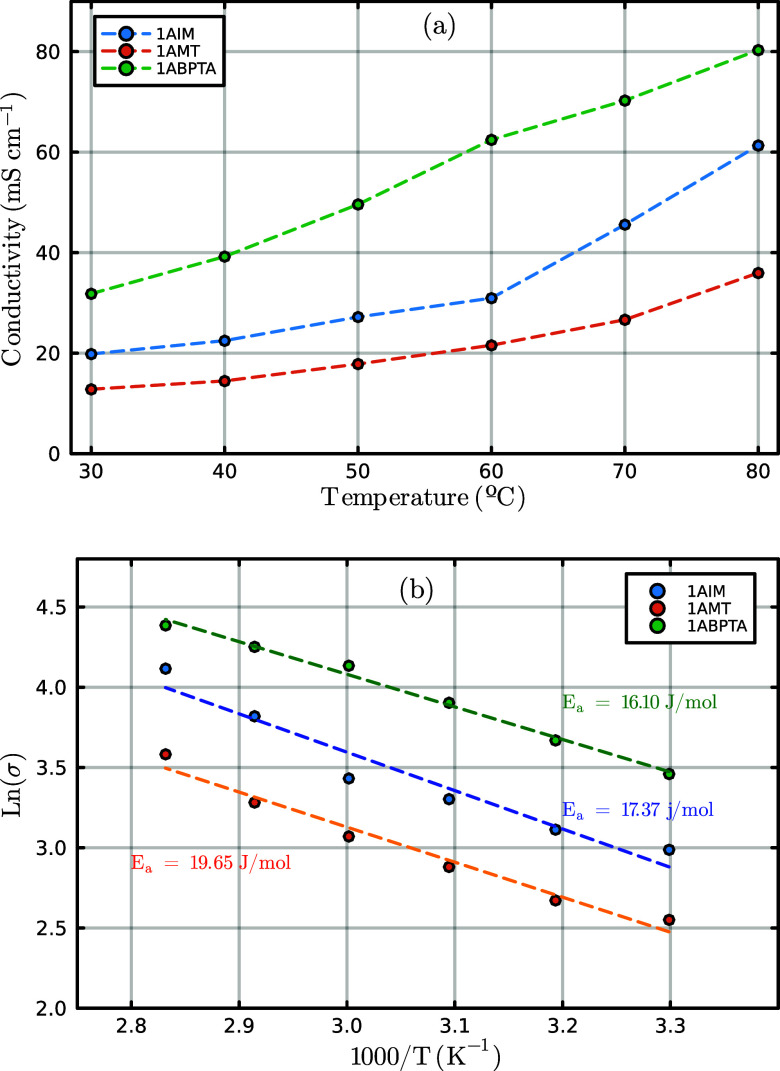
OH^–^ conductivity (a) and Arrhenius plots (b) for polycationic polymers.

An increase in all properties can be observed with the presence of more hydrophilic groups in the membrane; a greater number of cationic groups per monomer unit in 1ABPTA generate higher values of conductivity and WU. As expected, WU and SR increase with increasing temperatures. The 1ABPTA that exhibits the highest IEC value (2.84) presents the highest WU of 18.52 and 20.00% at 30 and 80 °C, respectively, while polymers that have a lower IEC, 1AIM and 1AMT, due to the presence of only a single formal charge per monomeric unit in their structure, display lower WU (11.11/19.05% and 12.05/16.67% at 30–80 °C, respectively). These values are lower in comparison with previously reported membranes^2^ with values of WU 26.7 and 27.7% for P(4PA-*co*-2PA)-Py and P(4PA-*co*-2PA)-Im polymers. These low values can be explained by the hydrophobic nature of the aromatic backbone. The presence of only *p*-terphenyl units in the main chain of the polymers promotes high hydrophobicity and more rigid chains. In fact, the *p*-terphenyl units confer low SR to the membranes and provide them with dimensional stability, which is fundamental to the highly preferable features for AEMs to be used in electrochemical devices. Hydroxide ion conductivity was measured at fully hydrated conditions with a variation of temperature from 30 to 80 °C. The ion conductivity of AEMs is mainly dependent on IEC and WU values; in this sense, the hydration number (λ) represents the number of water molecules per cationic group, being a direct measure relating both parameters. The fact that 1AMT exhibits a higher hydration number but lower conductivity may be associated with the formation of more than one hydration shell of water over the ionic sites. This phenomenon dilutes the concentration of charge carriers resulting in a decrease in conductivity.^53,54^ Membranes exhibit an increase in ion conductivity at higher temperatures with maximum values of 61.33, 35.97, and 80.33 mS cm^–1^ at 80 °C with hydration numbers of 5.88, 8.74, and 3.91 for 1AIM, 1AMT, and 1ABPTA, respectively. Figure 6 shows the –OH conductivity at different temperatures and the Arrhenius plots.

As expected, an enhancement of conductivity with the presence of more cationic groups was observed. The polymer 1ABPTA exhibited higher hydroxide conductivity compared to the polymers 1AIM and 1AMT, due to the presence of two cationic sites per monomer unit, and increased density of cationic sites facilitates the anion transport through the membrane, resulting in superior ionic conductivity. This effect is further evidenced by the activation energies for ion transport, derived from Arrhenius plots, Figure 6b, which show a decrease of over 1.27 J/mol and 3.55 J/mol in 1APBTA in comparison with 1AIM and 1AMT, respectively, indicating an ion transport thermodynamically more favorable. Although its WU values are not high, these poly((*p*-tolyl)imidazoleterphenylene) polymers have conductivities comparable with the most common commercial membranes like Aemion^55^ or Fumasep.^56^Table 5 shows some properties of different polymer membranes containing imidazolium groups. Compared with PIm-PBI, HIm-PBI, PAEK-APBI, and P-MeIM-tPh, the synthesized polymer 1ABPTA has higher hydroxide ion conductivity with a higher IEC value, except for the membrane PAP-IM-3,^57^ which presents the highest value of ion conductivity and IEC, but this is attributable to the difference in the backbone and to two distinct cationic groups (piperidinium and imidazolium).

**Table 5:** Properties of Some Imidazolium AEMs

membrane	IEC (mmol/g)	WU^b^ (%)	SR^b^ (%)	σ^b^ (mS cm^–1^)	reference
1AIM	1.88	12.77	12.74	61.33	this work
1ABPTA	2.84	15.63	10.52	80.31	this work
PIm-PBI	2.29	30.0 ± 1.8	11.2 ± 1.0	57.60	(58)
HIm-PBI	2.09	36.9 ± 3.7	15.7 ± 1.1	63.44	(58)
PAEK-APBI	1.43	≈45.0	≈16.0	≈35.0	(59)
P-MeIM-tPh	1.37	36.5^c^	11.3^c^	45.6	(29)
PAP-Im-3	2.96	59	12.4	165.3	(57)
Fumapem^a^	2.02	40^c^	-	40.0–45.0	

a Technical datasheet—Fumapem FAA-3–50.

b Measurement at 80 °C.

c Measurement at 30 °C.

**Figure 7:**
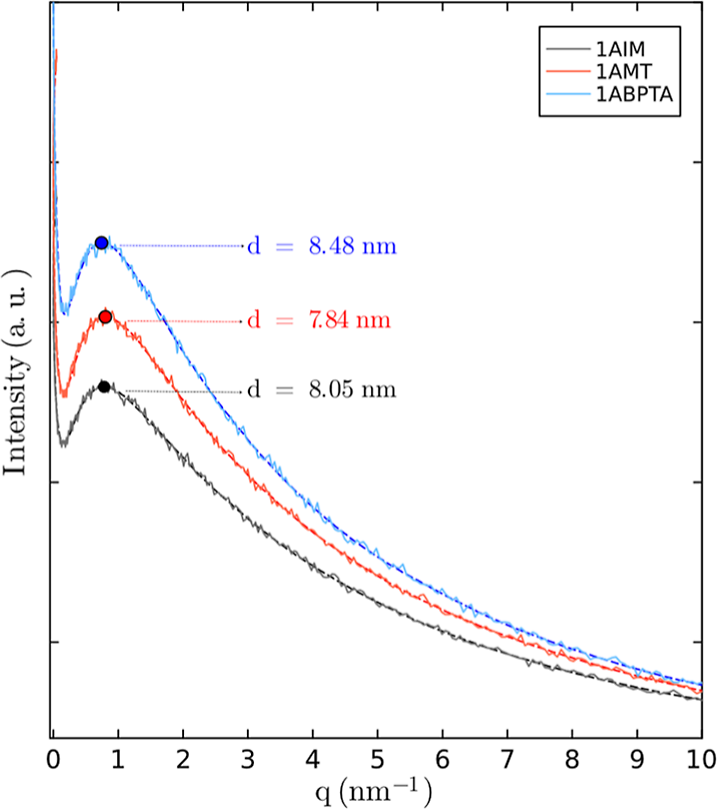
SAXS profiles of polyelectrolytes in bromide form.

A high ionic conductivity could be related to high IEC values, but the correct balance between hydration, WU, SR, and IEC remains a major drawback. As mentioned, high IEC values are related to high conductivities, but the mechanical properties are generally compromised due to the elevated values of WU and SR in the membranes; therefore, the efficiency of ion transport plays an important role. It has been established that a good hydrophilic/hydrophobic phase segregation favors ionic conductivity because conduction microchannels are generated. The microphase segregation of polyelectrolytes was studied via small-angle X-ray spectroscopy (SAXS) and atomic force microscopy (AFM). Figure 7 shows SAXS patterns, with ionomer peaks detected as expected. The curves indicate similar patterns across the three samples, suggesting a comparable morphology despite differences in the cationic groups and the length of the aliphatic chains. This indicates that the self-assembly of these groups into ionic clusters generates a common mesoscopic structure. The SAXS peaks correspond to the characteristic distance between the hydrophilic domains (ionic clusters), separated by interconnected ionic channels, reflecting the overall organization of the ionic clusters within the material.^60,61^

Bragg’s formula (*d* = 2π/*q*_max_) shows that the intercluster spacings between the samples are very similar (8.05, 7.84, and 8.48 nm for 1AIM, 1AMT, and 1ABPTA, respectively). This suggests that, despite differences in chain length, the organization and separation of the ionic clusters are comparable. Electrostatic interactions appear to dominate the formation of the ionic clusters and the connectivity of the hydrophilic domains, making variations in the structure of the cationic groups and chain length which does not significantly affect the mesoscopic morphology.

**Figure 8:**
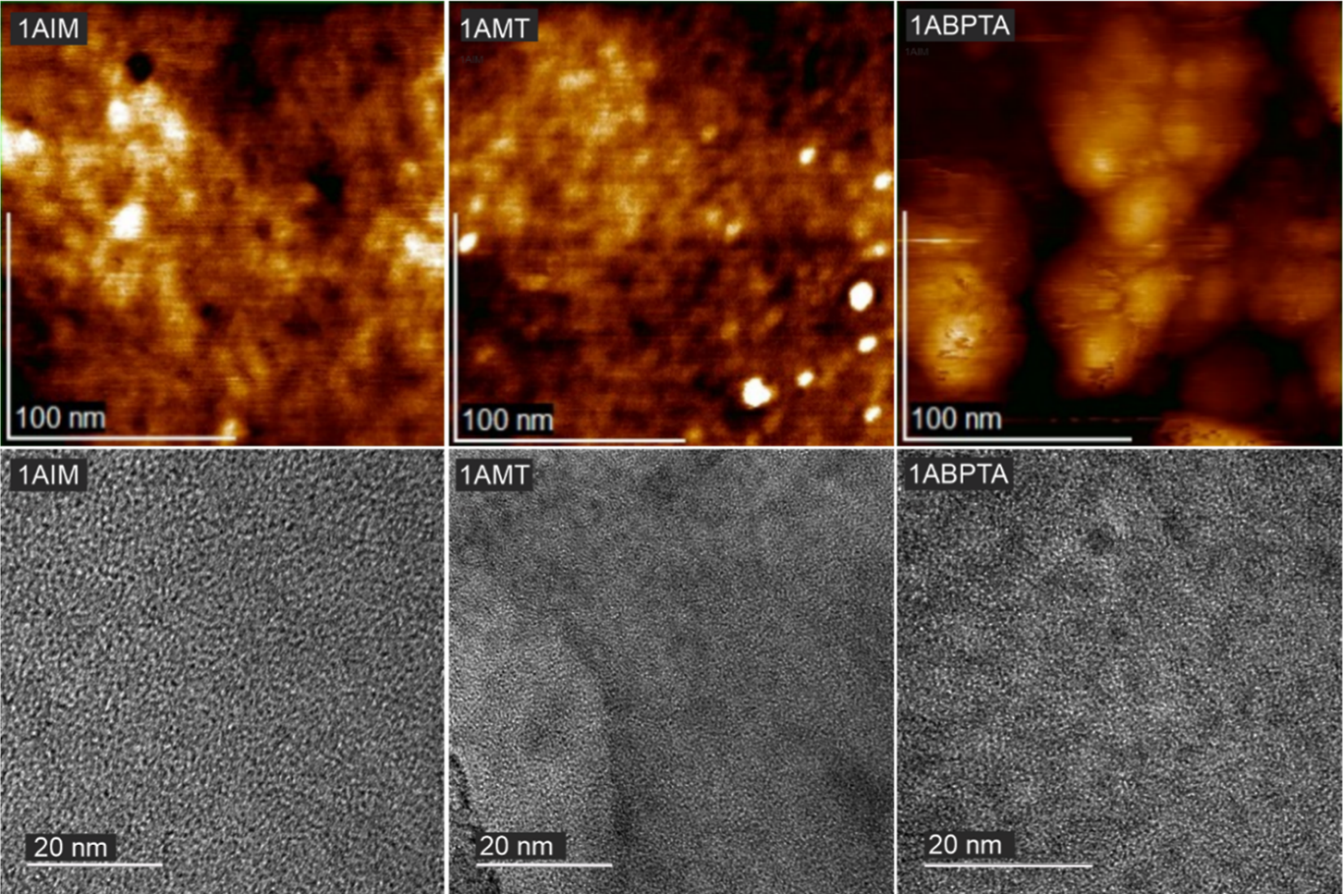
AFM topographical height image (top) and TEM images (bottom) of 1AIM, 1AMT, and 1ABPTA samples.

Figure 8 illustrates the AFM phase diagram (upper images), clearly depicting the distinction between light and dark regions corresponding to hydrophobic and hydrophilic domains on the polymer surface and ensuring phase segregation. This phase segregation results from the differences in charges and interactions between the components of the polymer. The hydrophilic regions contain cationic groups, such as quaternary ammonium groups in the case of 1ABPTA, which are attracted to water due to their positive charges, while the hydrophobic regions in the backbone polymers have no affinity with water. These segregated phases are crucial for the formation of ionic channels to facilitate the hydroxide ions conductivity.^62,63^

An increase in the aliphatic chain length and the number of ionic groups enhances the clarity of phase segregation, as depicted in Figure 8, as it favors a greater separation between the hydrophobic and hydrophilic phases. In the particular case of 1ABPTA, the distribution of the ionic clusters is approximately spherical. This ionic cluster, formed by the self-assembly of the cationic groups,^64^ is key in the development of the ion conducting pathways. The presence of these well-defined ionic clusters contributes to the formation of a more organized and efficient structure for the development of ionic channels, which could improve the material’s ionic conductivity.^65^ TEM confirms the presence of phase separation in the developed AEMs, as can be seen in Figure 8. The dark regions correspond to the hydrophilic imidazolium functional groups. On the other hand, the bright regions are related to the hydrophobic domains of the *p*-terphenyl units in the backbone. 1ABPTA presents a well-defined distribution of the hydrophilic domains, mainly due to its highest cation content, with an average size between 6 and 10 nm (Figure S5, TEM diffraction pattern), indicating that a nanophase separation microstructure between hydrophilic/hydrophobic domains was uniformly established. This correlates with the highest ion conduction for the developed materials in this work.

**Scheme 3:**
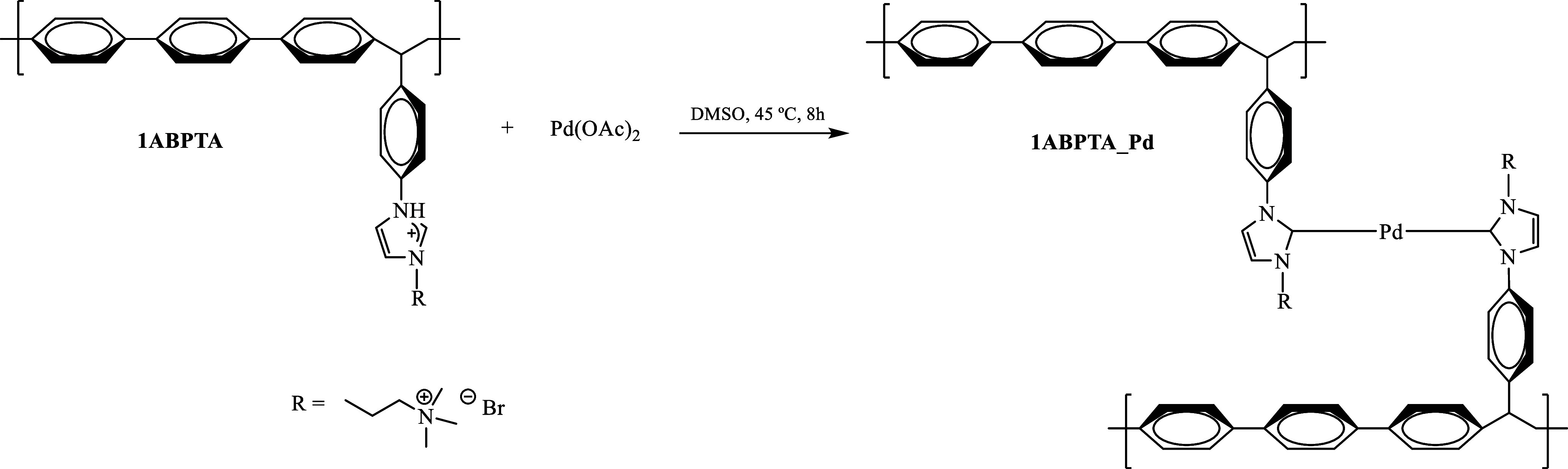
Synthesis of 1ABPTA with Palladium Acetate to Obtain an Organometallic Polymer

The presence of a *N*-heterocyclic ligand in the polymeric structure (imidazole group) broadens its usefulness in organometallic chemistry as an *N*-heterocyclic ligand due to its strong σ-donating characteristics and its ability to bind and stabilize a variety of transition metal complexes. These carbene complexes show high catalytic activities in several metal-mediated organic transformations such as C–C coupling reactions. One advantage of these carbenes complexes in comparison to the phosphine complexes is their stronger interaction between carbene donor function and the metal center giving them stability and the option of being reused as a catalyst.^66^ In this way, the use of a polyelectrolyte such as 1ABPTA promotes carbene formation by creating an ionic environment that stabilizes intermediate species and facilitates the deprotonation of the imidazole, thereby enhancing the generation of the reactive carbene center. This polymeric structure also helps disperse the carbene, preventing its deactivation through dimerization and improving its interaction with the metal. As a result, the resulting organometallic complex exhibits greater catalytic efficiency.^67^ Consequently, the product of the reaction between polyelectrolyte 1ABPTA and palladium acetate (Scheme 3) was tested in a Suzuki reaction to evaluate its catalytic performance. Stability and reaction conditions are summarized in Table 6.

**Table 6:** Reaction Results for the Organometallic Polymer

ID	stoichiometry	solvent	temperature (°C)	reaction time (h)	yield (%)
1ABPTA_Pd	1:0.7	DMSO	45	8	82

**Figure 9:**
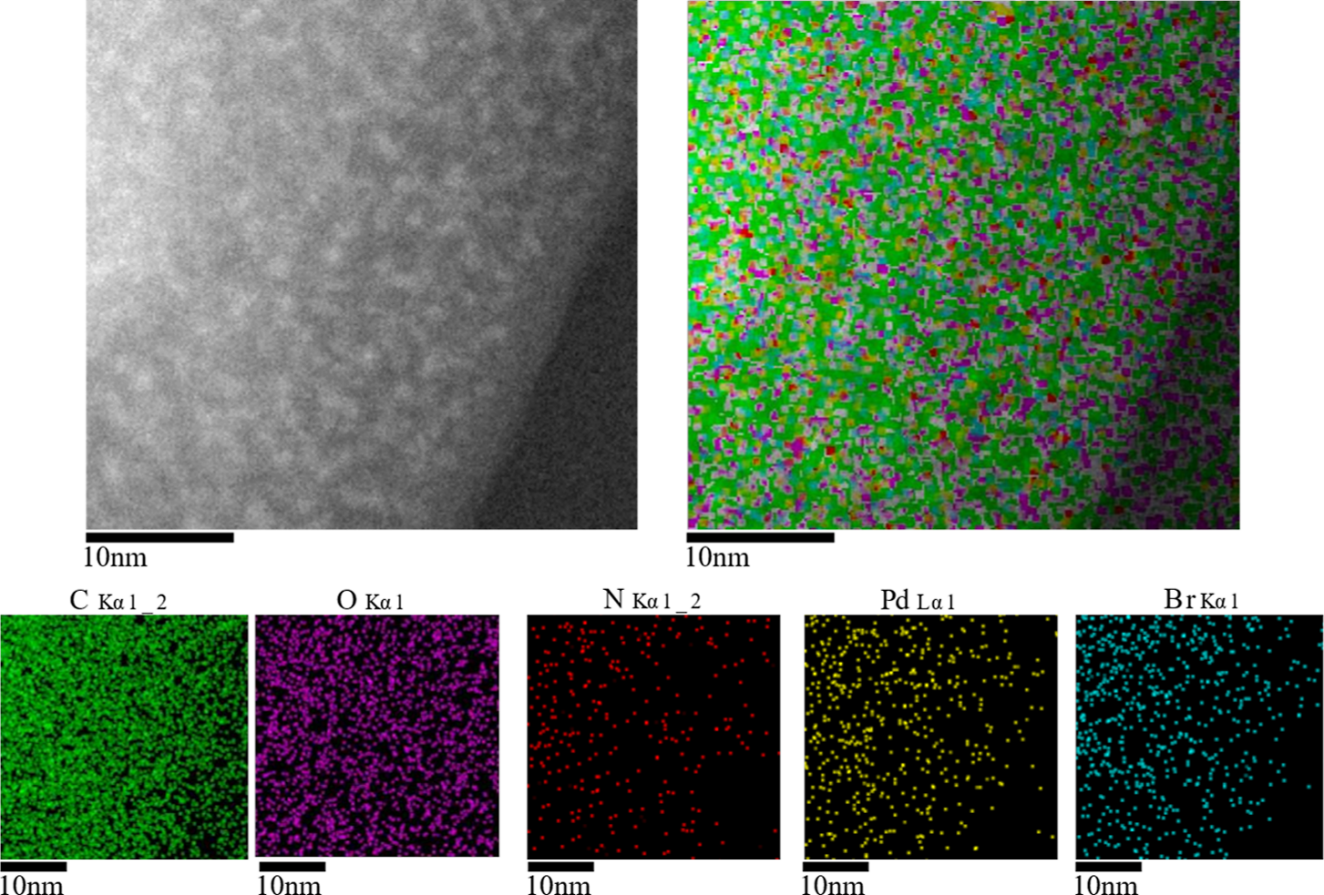
TEM images of the 1ABPTA_Pd polymer.

The growth of palladium nanoparticles in a polymer matrix has been reported by various authors.^68,69^ Depending on the reaction time and medium, the sizes of the nanoparticles obtained varies. However, the growth of these nanoparticles in a polymer matrix is not uniform; instead, it occurs in localized regions.^69,70^ Therefore, the obtained metal complex was analyzed by TEM. Figure 9 points toward a uniform distribution of palladium atoms rather than the formation of metal clusters in localized areas.

**Figure 10:**
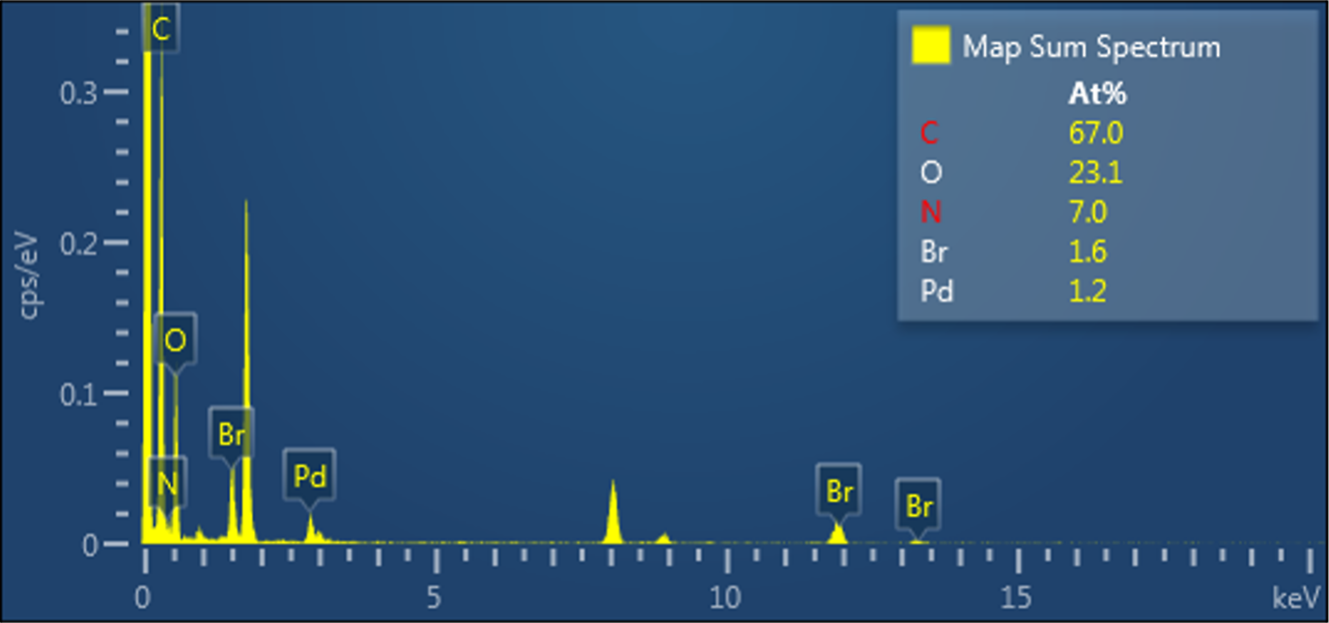
X-ray emission spectrum of the 1ABPTA_Pd polymer.

As expected, an elemental mapping shows the uniform distribution of Pd (yellow) and oxygen (purple) atoms, in percentages of 1.2% and 23.1%, respectively, belonging to the palladium incorporated in the polymer. These percentages were corroborated by EDX, Figure 10, showing a higher percentage of carbon atoms (67%) and to a lesser extent the presence of oxygen (23.1%), nitrogen (7.0%), bromine (1.6%), and palladium (1.2%).

**Figure 11:**
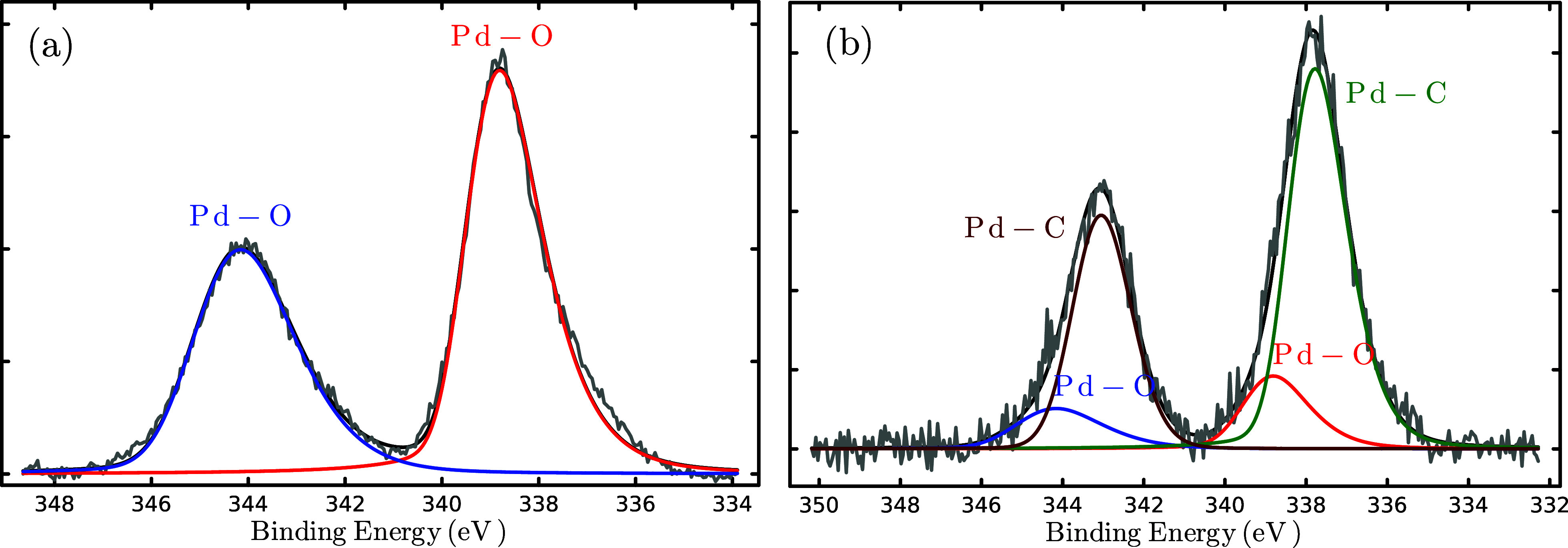
XPS spectra of palladium for (a) Pd(OAc)_2_ and (b) 1ABPTA_Pd.

To corroborate the formation of the C–Pd bond, XPS measurements were performed. Figure 11 displays the XPS spectra for the Pd 3d core level of Pd(OAc)_2_ and 1ABPTA_Pd. The binding energies (BE) of the functional groups are summarized in Table 7. The Pd 3d core level spectra for palladium acetate (Figure 11a) indicate a single valence state corresponding to Pd(II) for the Pd–O bond in Pd^II^3d_3/2_ (BE = 344.16 eV) and Pd^II^3d_5/2_ (BE = 338.81 eV). As for the organometallic polymer, a shift in bond energies in the Pd 3d core level spectra for 1ABPTA_Pd (Figure 10b) was observed, indicative of the formation of the Pd–C bond for Pd^II^3d_3/2_ (BE = 343.05 eV) and Pd^II^3d_5/2_ (BE = 337.78 eV), alongside residual Pd–O bonding. The presence of palladium nanoparticles is ruled out because no peak corresponding to zerovalent palladium (Pd(0)) was observed at 335.5 eV.^71,72^ Furthermore, the C 1s and N 1s spectra between 1ABPTA and 1ABPTA_Pd display shifts associated with the C–Pd and N–C–Pd bonds, as can see in Figures S3 and S4, respectively.^73,74^

**Table 7:** BE and Percentages of Different Bonds Obtained from XPS Spectral Deconvolutions

	palladium bonds		
system	bond	BE (eV)	at %^a^
1ABPTA_Pd	Pd–O (Pd^II^3d_3/2_)	344.16	7.9
	Pd–O (Pd^II^3d_5/2_)	338.81	11.2
	Pd–C (Pd^II^3d_3/2_)	343.05	29.7
	Pd–C (Pd^II^3d_5/2_)	337.78	51.2
Pd(OAc)_2_	Pd–O (Pd^II^3d_3/2_)	344.16	41.6
	Pd–O (Pd^II^3d_5/2_)	338.81	58.4
Pd^0^	Pd^0^3d_5/2_	335.2^59^–335.5^62^	-

a Contributions percentage of different bonds at the core level peak.

**Scheme 4:**
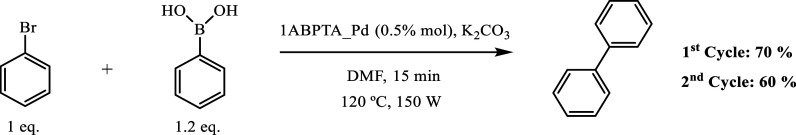
Evaluation of the Catalytic Activity of the 1ABPBA_Pd Complex

The elemental analysis obtained from the XPS spectra (0.52% O, 62.67% C, 5.51% N, 14.89% Br, and 16.41% Pd wt %), removing the adventitious carbon, evidenced that all the polymer present is coordinated with palladium, as shown in Scheme 3. Approximately 4.4% of the palladium present (26.7% of the total palladium) is in the form of palladium oxide (PdO) as a byproduct of the precursor salt. The catalytic activity of the obtained polymer–metal complex 1ABPTA-Pd was evaluated on a Suzuki–Miyaura coupling reaction (Scheme 4) using a catalyst loading of 0.5 mol % at 120 °C for 15 min. The tests showed a conversion yield of 70% for the first catalytic cycle and 60% conversion for the second catalytic cycle. The results obtained were encouraging as compared to the results obtained by Karatas et al.^75^ where a 32% conversion using anthracene-substituted imidazole-based palladium *N*-heterocyclic carbene complexes with a catalyst loading of 2 mol % at 80 °C for 2 h was observed or the report of Lambert et al.^76^ with a 35% conversion using the (NHC)_2_Cl_2_ discrete Pd complex with a catalyst loading of 0.15 mol % at 110 °C for 1 h.

The synthesis of the organometallic polymer maintains the charge on the side chain due to the presence of the quaternary ammonium group. The main difference of these organometallic polyelectrolytes in comparison with neutral organometallic compounds results in higher thermal and chemical stability. The incorporation of metal centers enhances resistance to degradation in alkaline media, preventing elimination or hydrolysis processes typical of quaternary ammonium or imidazolium groups.^77^ Additionally, these materials exhibit thermal stability above 300 °C and remarkable resistance to redox processes, making them suitable for high-temperature, electrochemical, and catalytic applications.

## 4 Conclusions

Novel polyelectrolytes were synthesized through a polycondensation reaction of *p*-terphenyl and 4-(1*H*-imidazol-1-yl)benzaldehyde in a superacidic medium, followed by efficient modification with different side chains. The materials obtained were cast into membranes exhibiting distinctive properties such as excellent mechanical and thermal properties due to the absence of ether linkages in the structure. The membranes showed channels for ion transport due to the separation of hydrophilic/hydrophobic microphases, resulting in high conductivities, 61.33 and 80.33 mS/cm at 80 °C for 1AIM and 1ABPTA, respectively. The presence of an imidazolium group pendant on the main polymeric chain allowed the reaction with palladium acetate to obtain an organometallic complex 1ABPTA_Pd. Catalytic activity of the complex was tested in the Suzuki–Miyaura C–C coupling reaction, yielding 70% and 60% for the first and second cycles, respectively. These properties evidence the potential use of these polyelectrolytes in electrochemical devices and catalysis, offering multipurpose functionalities for further exploration.

## Supporting Information Available

Additional ^1^H and 2D NMR spectra and C 1s and N 1s core level XPS (PDF)
