# Supplementary material for: Functionalized Imidazolium Ether-Free Polymer Backbones with Ion Transport Channels and Catalytic Activity
Source: ACS Mater Au. 2025 Mar 27;5(3):508–21. doi: 10.1021/acsmaterialsau.4c00154 (PMC12082359; doi:10.1021/acsmaterialsau.4c00154)
Supplement: Supplementary file 1 — mg4c00154_si_001.pdf [file mg4c00154_si_001.pdf]

Supporting information

Functionalized Imidazolium Ether-Free Polymer Backbones with Ion Transport Channels and Catalytic Activity

Bryan A. Corzo[1], Hugo Hernández-Martínez[1], Eugenia Josefina Aldeco-Pérez[2], Jorge Cárdenas[3], Víctor Lara[4], Lilian I. Olvera[1*]

[1]Instituto de Investigaciones en Materiales, Universidad Nacional Autónoma de México, Apartado postal 70-360, CU, Coyoacán 04510, Ciudad de México, México.
[2]Centro de Investigación y Desarrollo Tecnológico en Electroquímica S.C., Parque Tecnológico Querétaro, C.P. 76703, Sanfandila, Pedro Escobedo, Qro, México.
[3]Instituto de Química, Universidad Nacional Autónoma de México, Apartado Postal 70-360, CU, Coyoacán, 04510, Ciudad de México, México.
[4] UAM-I, Av. Michoacán y Purísima, Iztapalapa, 09340, México City, México.

Supplementary spectra

The characterization of cross-peaks using a pair of coordinates in the 2D COSY ^{1}H NMR spectrum allows for a more unequivocal assignment of resonance peaks compared to the 1D ^{1}H NMR spectrum. This approach clarifies the assignment of aromatic proton peaks in the 7 - 8 ppm region, as shown in Figure S1.

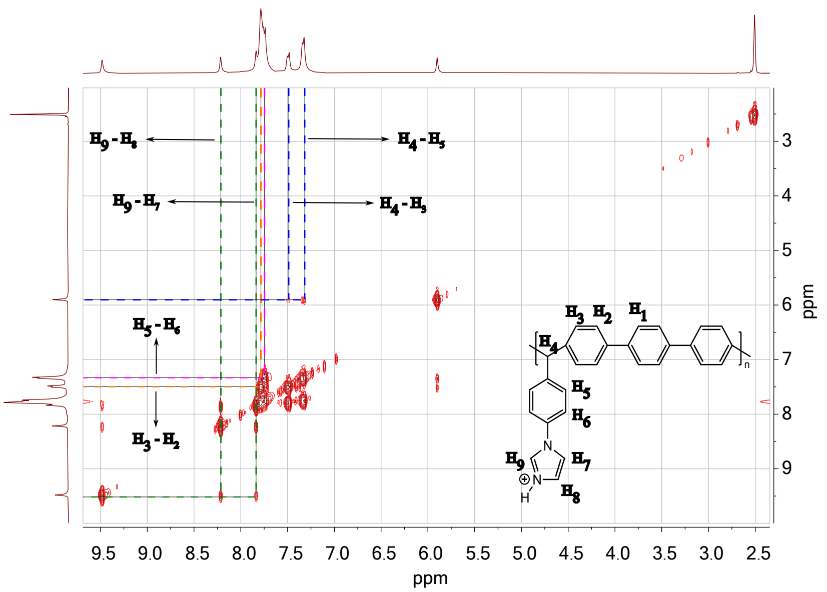

Figure S1. 2D COSY ^{1}H NMR spectrum of protonated base polymer $1A^+$.

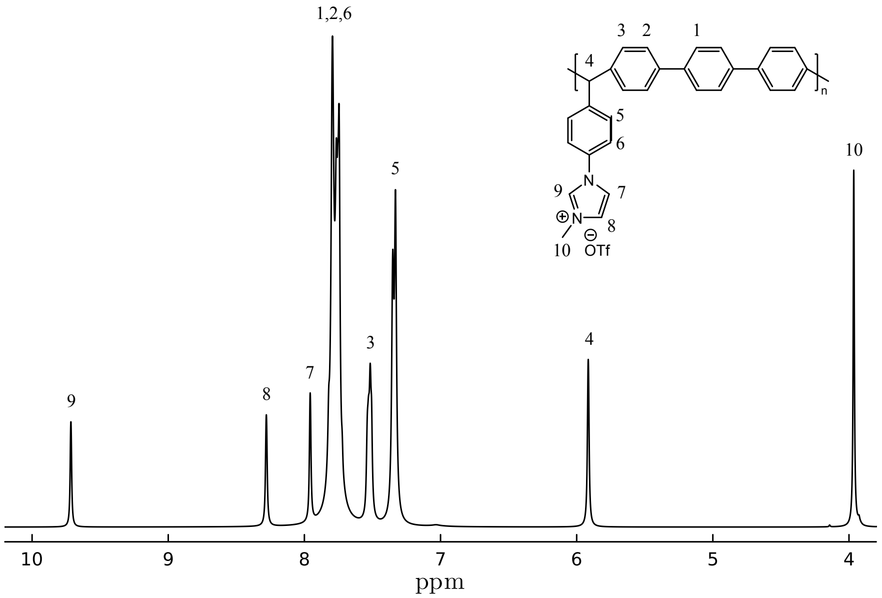

Figure S2. ^{1}H NMR spectrum of polyelectrolyte PMT.

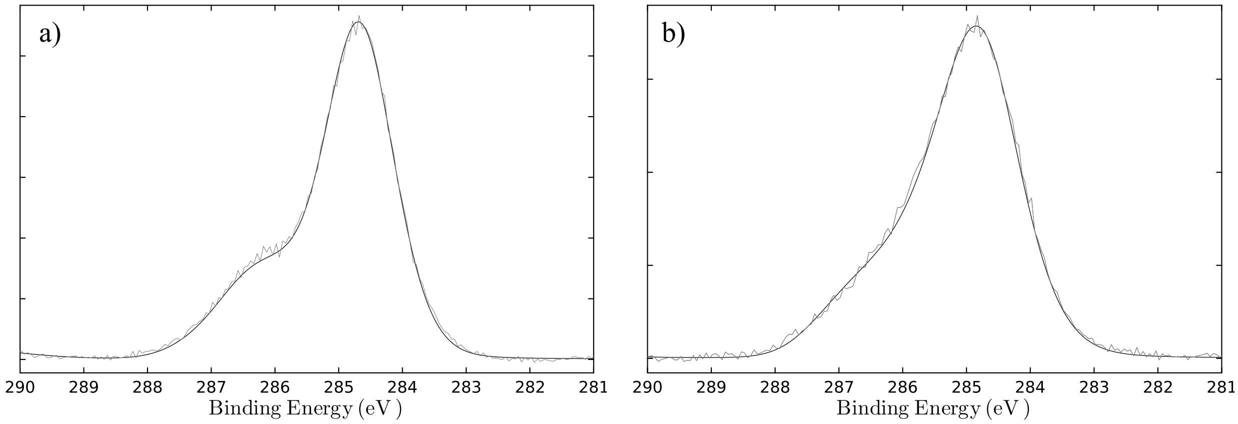

Figure S3. C1s core level XPS spectra for (a) PBPTA and (b) PBPTA_Pd.

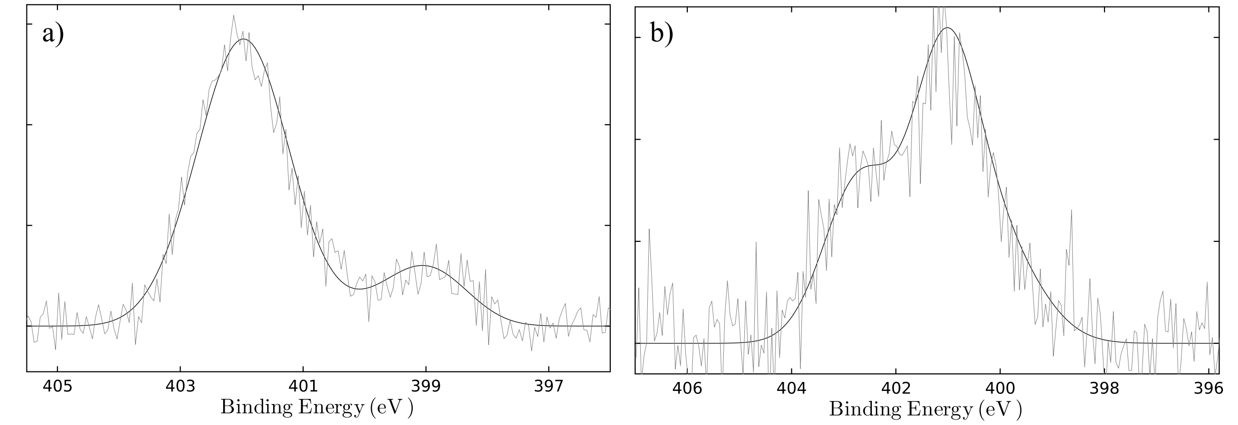

Figure S4. N 1s core level XPS spectra for (a) PBPTA and (b) PBPTA_Pd.

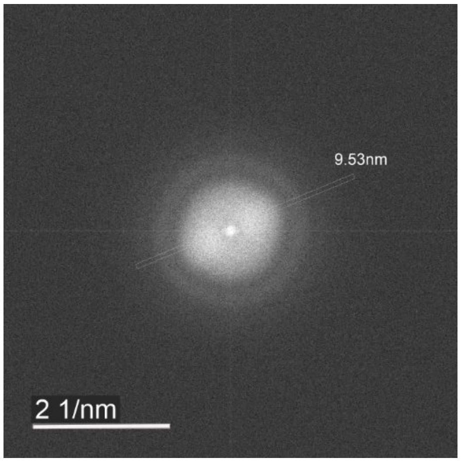

Figure S5. TEM diffraction pattern of AEMs
